# Exploring drought-responsive crucial genes in *Sorghum*

**DOI:** 10.1016/j.isci.2022.105347

**Published:** 2022-10-14

**Authors:** Yilin Bi, Pei Wang

**Affiliations:** 1 School of Mathematics and Statistics, Henan University, Kaifeng 475004, China; 2 Center for Applied Mathematics of Henan Province, Henan University, Kaifeng 475004, China

**Keywords:** Natural resources, Genetics, Plant biology, Agricultural science

## Abstract

Drought severely affects global food production. *Sorghum* is a typical drought-resistant model crop. Based on RNA-seq data for *Sorghum* with multiple time points and the gray correlation coefficient, this paper firstly selects candidate genes via mean variance test and constructs weighted gene differential co-expression networks (WGDCNs); then, based on guilt-by-rewiring principle, the WGDCNs and the hidden Markov random field model, drought-responsive crucial genes are identified for five developmental stages respectively. Enrichment and sequence alignment analysis reveal that the screened genes may play critical functional roles in drought responsiveness. A multilayer differential co-expression network for the screened genes reveals that *Sorghum* is very sensitive to pre-flowering drought. Furthermore, a crucial gene regulatory module is established, which regulates drought responsiveness via plant hormone signal transduction, MAPK cascades, and transcriptional regulations. The proposed method can well excavate crucial genes through RNA-seq data, which have implications in breeding of new varieties with improved drought tolerance.

## Highlights

• We design a method that unites gene rewiring network and Markov random field model
• Drought-responsive genes for five developmental stages of *Sorghum* are explored
• A multilayer network reveals that *Sorghum* is very sensitive to pre-flowering drought
• A drought-responsive crucial gene regulatory module is established for *Sorghum*

## Graphical abstract

**Figure:**
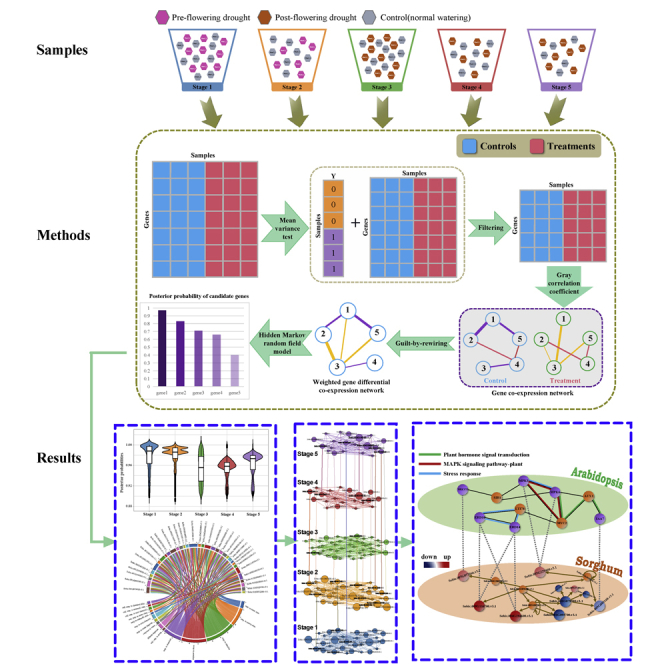

## Introduction

With the increasing of global population, food security has become a serious global problem. Drought is a typical abiotic stress that severely affects food security. It is reported that the impact of drought on crops is grievous among all abiotic stresses (Fahad et al., 2017; Jaiswal et al., 2021). It is estimated that drought can directly cause averagely $2.9 billion losses annually (Fahad et al., 2017). An efficient way to guarantee food security is to optimize and cultivate crops that can adapt quickly to environmental changes (Council, 1996), such as drought stress. However, exploring drought-resistant mechanisms of crops and the associated crucial genes is the first step to cultivate novel drought-resistant varieties and alleviate the impact of drought on crop yields.

It is well known that different crops have varied water demand to maintain growth and development. Comparing with *Corn*, *Barley*, and *Wheat*, *Sorghum* is extremely resistant to drought, which can survive for several weeks without water (House, 1985). Actually, *Sorghum* is characterized by its low water consumption, high water utilization, and high photosynthetic efficiency; thus, it is widely planted in arid and semi-arid areas, and it has become an ideal plant for probing drought responsiveness (Mace et al., 2013). The genome of *Sorghum* was firstly published in 2009 (Paterson et al., 2009), and considerable research on transcriptomic sequencing have been subsequently performed (Mace et al., 2013; Varoquaux et al., 2019; Zhang et al., 2019), which facilitate us to systematically explore its drought-resistant mechanisms from omics data (Ngara et al., 2021).

For omics data analysis, a challenge issue is to develop appropriate mathematical and statistical tools to explore useful bioinformatics. Various data-driven techniques have been developed and great advances have been made during the last decades. The associated techniques include dimensional reduction and variable pre-selection, network reconstruction and network-based information mining, sophisticated model-based methods for crucial gene identification, and so on (Lü and Wang, 2020).

Hereinafter, we briefly review some related works on omics data analysis. First of all, massive omics data often contain too many covariates but with only a few samples, considerable actually uncorrelated or independent covariates greatly hinder the subsequent analysis and applications (Wang et al., 2022). Therefore, it is necessary to perform dimensional reduction or variable pre-filtering. RNA-seq data often include samples for treatments and controls. In the experimental design, a discrete categorical response variable can be representative of the experimental environment in which the samples are placed, and expression profiles of genes can be considered as covariates. Certainly, it is also possible to consider some informative genes of interest as response variables. In practice, the independence test between the response variables and the covariates can be used to exclude irrelevant variables. Many methods have been developed to perform the independence test. For example, the Kolmogorov-Smirnov test is a nonparametric method to test whether the distribution of a sample is consistent with another, but it is only suitable for continuous quantitative data (Lilliefors, 1967). The Pearson $\chi^{2}$ test discretizes the continuous random variables to check the independence between two variables, but it may lead to information loss due to the discretization processes (Dahiya and Gurland, 1972). The two-sample *t* test is only applicable to continuous variables, and it needs to assume that samples follow normal distributions (Xu et al., 2017). The recently proposed model-free mean variance (MV) test can be used to detect the independence between a continuous random variable and a categorical variable (Cui and Zhong, 2019). Except the mentioned independent tests, many other methods can also be used to realize dimensional reduction and variable selection (Moore, 2004; Hahn et al., 2003; Macciotta et al., 2009). The associated methods can help researchers to effectively exclude non-critical variables and reduce computational burden.

Secondly, complex network has been widely used to explore omics data (Lü et al., 2012, 2016; Kawata et al., 2018; Ding et al., 2020; Shang and Liu, 2021; Liu et al., 2012, 2014; Csermely et al., 2013). For example, various network-based approaches have been developed to explore drug targets and essential proteins (Csermely et al., 2013; Shang and Liu, 2021; Liu et al., 2012, 2014), as well as stress-responsive crucial genes in plants (Wang et al., 2017; Wang et al., 2018a; Wang et al., 2018b; Wang, 2021; Bi and Wang, 2022). Recently, Wang et al. (Wang and Wang, 2022) proposed a novel method to construct gene differential co-expression networks (GDCNs), and then based on the GDCNs, they developed three indexes to evaluate the importance of genes in altering global co-expression patterns. The network-based approach provides effective tools to explore omics data.

Finally, as to model-based methods for crucial gene identification, many methods or algorithms have been reported, including the hidden Markov random field (HMRF) model (Besag, 1986). Generally, HMRF model can be used to describe the noncausal context relationship or spatial relations in physical phenomena. The HMRF model has been widely used in genome-wide association studies (GWAS). For example, based on the guilt-by-association principle (Xu and Li, 2006; Wu et al., 2008; Jeong et al., 2001), Chen et al. incorporated biological pathway information into the HMRF model to screen informative GWAS signals (Chen et al., 2011). However, Chen et al. overlooked the dynamic feature of biological networks (Wu et al., 2008; Yang et al., 2011; Vanunu et al., 2010; Chen et al., 2009; Lee et al., 2011). Subsequently, Hou et al. integrated gene rewiring networks into the HMRF to study the Crohn and Parkinson diseases. They introduced the guilt-by-rewiring principle in the HMRF model to prioritizing genes. The method proposed by Hou et al. considered the dynamic characteristics of the networks (Hou et al., 2014), which is biologically meaningful. However, the existing models need to integrate multiple omics data, including gene expression data and GWAS data, which are inappropriate for the cases without required data. Moreover, the use of multiple omics data unavoidably introduces bias and batch effect, which inspire us to develop novel methods that merely rely on single omics data, such as RNA-seq data.

Motivated by the mentioned issues, we will explore the RNA-seq data for *Sorghum* under drought stress and with multiple time points. Firstly, the MV test is used to exclude genes that are independent with the response or phenotype; then, based on the expression data of the selected genes under treatments and controls and the gray correlation coefficient (GCC), weighted gene differential co-expression networks (WGDCNs) are constructed. Finally, combining the WGDCNs and the HMRF model, the posterior probabilities of genes that contribute to drought stress are obtained. GO enrichment analysis and gene sequence alignment analysis reveal that the screened crucial genes play critical functional roles during drought stress in *Sorghum*. The main contribution of this paper includes three aspects: 1) A method that integrates the WGDCN and the HMRF model is proposed to analyze RNA-seq data, which has the advantages of both considering the network structural information and the sophisticated statistical model; 2) The RNA-seq data for *Sorghum* under drought stress and with multiple time points are explored; drought-responsive crucial genes are identified for different developmental stages, and their biological functions are investigated in detail; 3) A multilayer differential co-expression network and a possible gene regulatory module are established, which can be used to reveal certain mechanisms of drought responsiveness in *Sorghum*.

## Results

### Method summary

**Figure 1:**
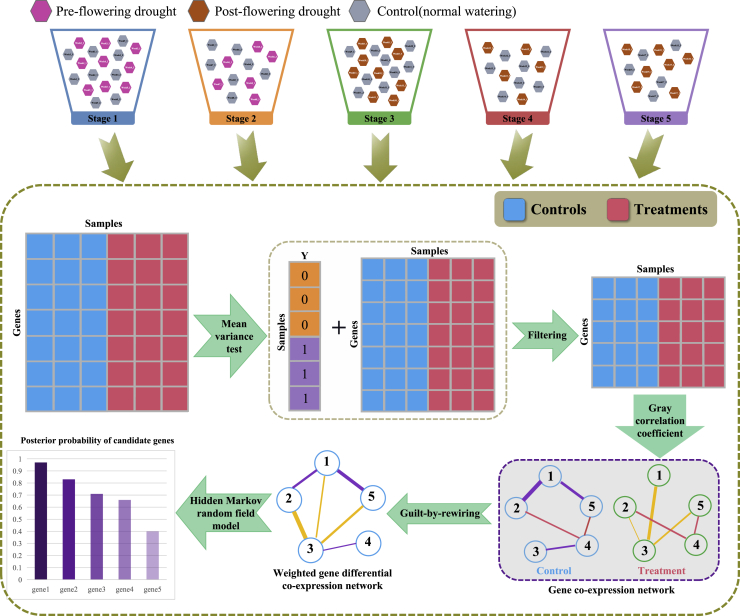
Schematic flowchart of the proposed method to identify drought-responsive crucial genes in *Sorghum* RNA-seq data under pre-flowering drought, post-flowering drought and normal watering conditions are considered, and these samples are divided into five developmental stages. Each stage covers samples from three successive weeks. The notation week i_j stands for the sample of the *j*’th replicate at the *i*’th week. For each stage, the original RNA-seq data is firstly processed and filtered by the mean variance test; then based on the gray correlation coefficient and the guilt-by-rewiring principle, a weighted gene differential co-expression network is constructed. Finally, based on the weighted gene differential co-expression network and the hidden Markov random field model, posterior probabilities for candidate genes are obtained. Drought-responsive crucial genes are genes with high posterior probabilities.

Our goal is to statistically identify drought-responsive crucial genes in *Sorghum*. A summary of the proposed approach is depicted in Figure 1. Firstly, since *Sorghum* suffers great phenotypical changes from week 3 to week 17, and to be more concretely screen crucial genes at different developmental periods, we divide the RNA-seq data into five stages, each stage covers three weeks. This classification mainly considers the developmental features (Vanderlip and Reeves, 1972) of *Sorghum* and the balance of samples for each stage. Secondly, for the processed data from each stage, we perform MV test to exclude genes that are independent with treatments, and retain genes with $P_{MV}\leq 0.01$ as candidate genes for statistical analysis. Based on RNA-seq data of the selected genes, WGDCN is constructed for each stage. Finally, combining the WGDCN and the HMRF model (Method details), posterior probability of each candidate gene is obtained for each stage. The obtained posterior probability reflects the association tendentiousness of a gene with drought stress. The candidate genes can be prioritized according to the posterior probabilities, and the top-ranked genes are deemed as crucial ones.

### Rankings according to the proposed method have high discrimination ability

**Figure 2:**
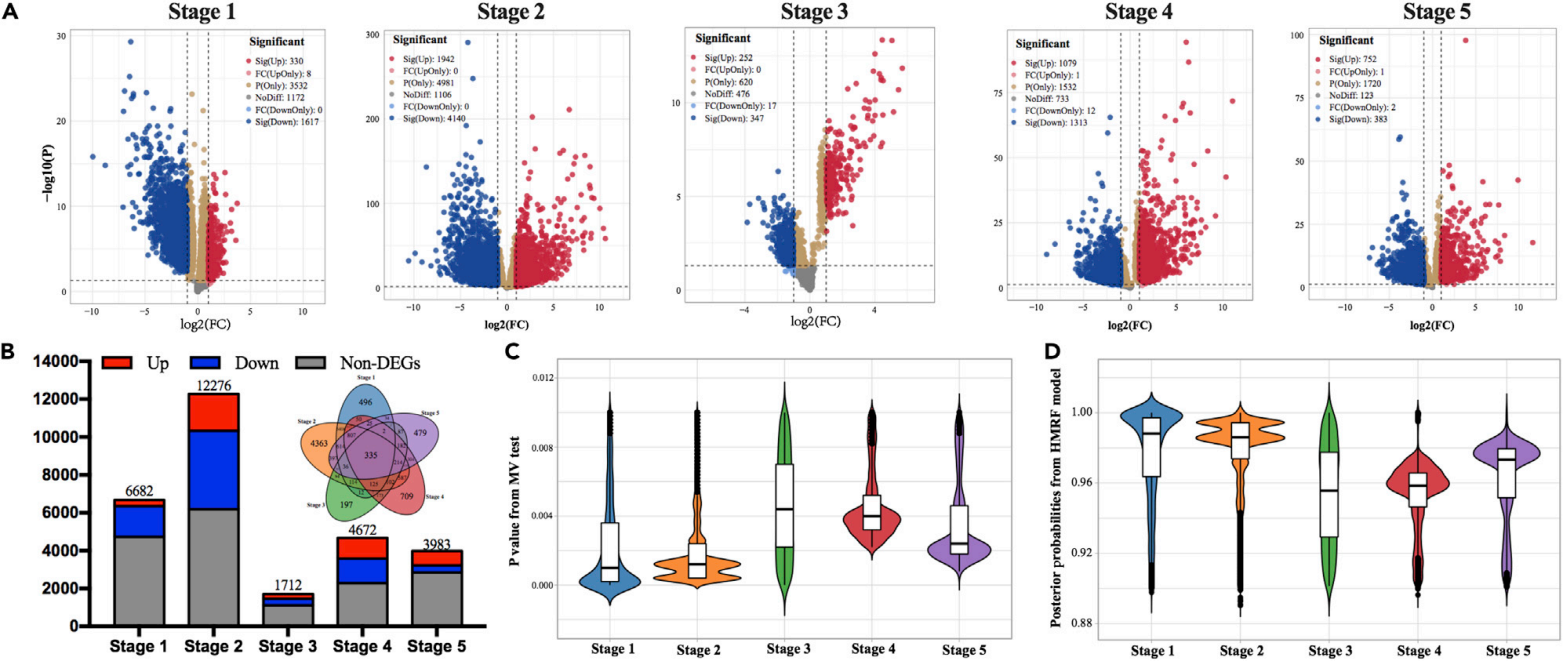
Information for candidate genes at the five stages (A) Volcano plots for candidate genes at each stage. sig(Up/Down) represents significantly up-/down regulated genes; FC denotes fold change of gene expressions between treatment and control; FC(Up/DownOnly) represents genes with $log_{2}\left(FC\right)\geq 1$ or $log_{2}\left(FC\right)\leq -1$, but their expressions are not significantly different between treatments and controls (P≥0.05). P(Only) denotes genes with P<0.05 and $|log_{2}\left(FC\right)|<1$; NoDiff denotes genes with both P≥0.05 and $|log_{2}\left(FC\right)|<1$. (B) The number of candidate genes at each stage and the corresponding Venn diagram. (C) The distributions of P values according to the MV test. (D) The distributions of posterior probabilities that obtained by the HMRF model.

Based on the RNA-seq data of *Sorghum*, the MV test screens 6682, 12,276, 1712, 4672, and 3983 genes at the five stages, respectively, where 335 genes are commonly selected at the five stages (Figure 2). In the former four stages, more differentially expressed genes (DEGs) are downregulated; whereas, at Stage 5, upregulated DEGs are more than the downregulated ones. About half of the candidate genes are not differentially expressed ($|log_{2}\left(FC\right)|<1$ or P>0.05). By incorporating the WGDCNs and the HMRF model, the posterior probabilities of the candidate genes are obtained. Our results reveal that considerable genes are with posterior probabilities ranging from 0.96 to 1, and there are slight differences between different stages (Figures 2C and 2D). The distributions of $P_{MV}$ (Figure 2C) and posterior probabilities (Figure 2D) show reverse trends, and there are some differences between the two, especially for the last two stages.

In order to compare the performance of the MV test and the HMRF model on their distinguish abilities, we define discrimination abilities of the MV test and the HMRF model at the *l*’th stage as

$$R_{l}^{MV}=\frac{U_{l}^{MV}}{m_{l}},R_{l}^{HMRF}=\frac{U_{l}^{HMRF}}{m_{l}}\left(l=1,2,3,4,5\right).$$

Here, $m_{l}$ is the number of candidate genes at Stage *l*, $U_{l}^{MV}$ is the number of unique rankings according to the MV test, and $U_{l}^{HMRF}$ denotes the number of unique rankings from the HMRF model at the *l*’th stage. Table 1 shows that the discrimination abilities of the MV test are apparently lower than those from the HMRF model, which indicates that the HMRF model can more precisely distinguish the differences among genes.

**Table 1:** Discrimination abilities of the MV test and the HMRF model at each stage. The rankings from the HMRF model have apparent higher discrimination abilities than those from the MV test

	Stage 1 (Week 3–5)	Stage 2 (Week 6–8)	Stage 3 (Week 9–11)	Stage 4 (Week 12–14)	Stage 5 (Week 15–17)
$R^{MV}$	0.0076	0.0038	0.0298	0.0086	0.0115
$R^{HMRF}$	0.7785	0.8146	0.9854	1.0000	1.0000

### Drought-responsive crucial genes and their functional analysis

Hereinafter, the top-20 ranked genes with high posterior probabilities at each developmental stage will be selected as crucial drought-responsive ones. The top-20 ranked genes account for ∼0.09% of all detected genes in RNA-seq.

**Figure 3:**
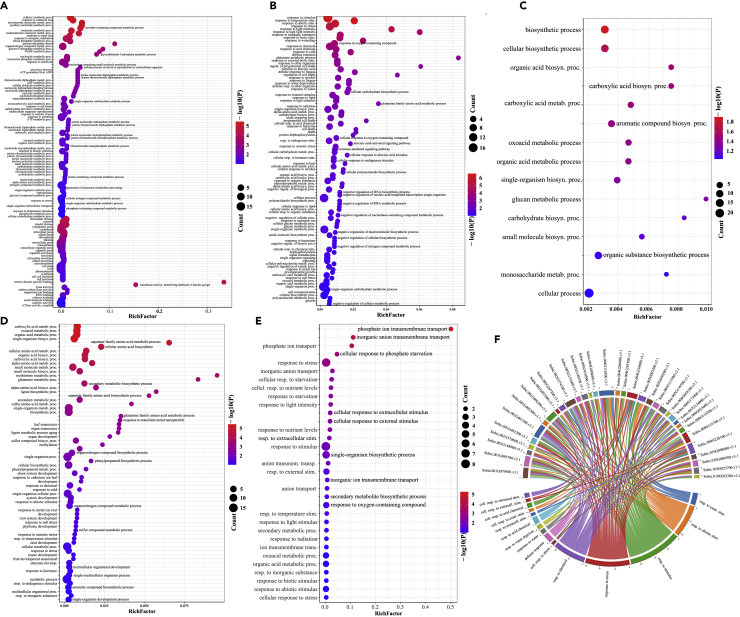
GO enrichment analysis for the top-20 ranked genes at the five stages (A–E) GO enrichment analysis for the top-20 ranked genes at each of the five stages. Enriched biological processes (P<0.1) for the top-20 ranked genes are considered. (F) Enriched drought-related processes and the associated top-20 ranked genes.

It is known that plants can cope with drought through various ways, such as metabolism (Bhargava and Sawant, 2012; Pinheiro and Chaves, 2011), biosynthesis (Capell et al., 2004; Ilhan et al., 2015), osmotic adjustment (Babita et al., 2010; Flowers and Yeo, 1986), stomatal closure, and reduction of photosynthetic rates (Pezeshki and Chambers, 1986). GO enrichment analysis reveals that the top-20 ranked genes are enriched in drought-related biological processes (Figures 3A–3E), including response to stimulus, response to stress (drought and oxidative stresses), and response to chemical. Figure 3F shows 15 enriched biological processes and the associated candidate genes. The 15 processes include response to stress/stimulus, regulation of response to watering, and cellular response to water deprivation. Among the associated genes, Sobic.001G0401300.v3.1 and Sobic.004G116300.v3.1 participate in many of the 15 biological processes; Sobic.001G079500.v3.1, Sobic.001G095700.v3.1, and Sobic.009G116700.v3.1 involve in responding to water and water deprivation. However, the GO enrichment results for the bottom-20 ranked genes are quite different from the top-20 ranked ones; no apparent processes are associated with drought responsiveness (Figure S1). GO enrichment analysis suggests that the top-20 ranked genes by the HMRF may actually play a key role during drought responsiveness in *Sorghum*.

Among the identified crucial genes, based on sequence alignment analysis (Johnson et al., 2008) with the *Arabidopsis* genome, we find that many genes are homologous with known drought-related genes in *Arabidopsis*, for example, Sobic.003G229400.v3.1 is possibly homologous with MPK3 and MPK6 (Table S1). Many studies have reported that MPKs play roles in regulating developmental processes and in responding to various stimuli in plants (Ma et al., 2017). Tsugama et al. reported that MPK6 can be directly regulated by drought, and ROS-induced MPK6 activation served as an upstream signal under drought conditions (Tsugama et al., 2012). Sobic.007G077466.v3.1 is homologous with WRKY66 and WRKY75, which belongs to the WRKY transcription factor (TF) family. The WRKYs play important roles during stress responsiveness in plants (Wang et al., 2018a). Some other homologous genes in *Arabidopsis* include PDC1, PMH1, LEA, SOS6, IAA7, PBS1, ARSK1, ERD14, RBOHD, and so on. Many of them involve in drought-related biological processes (including responding to water/water deprivation and cellular response to water deprivation), and partly of them have been proved by previous studies (Table S1).

As a summary, GO enrichment analysis and sequence alignment analysis with the *Arabidopsis* genome reveal that the identified top-20 ranked genes are inextricably associated with drought stress, which indicates that the proposed method is efficient in identifying crucial drought-responsive genes in *Sorghum*.

### Multilayer differential co-expression network analysis for the identified crucial genes

**Figure 4:**
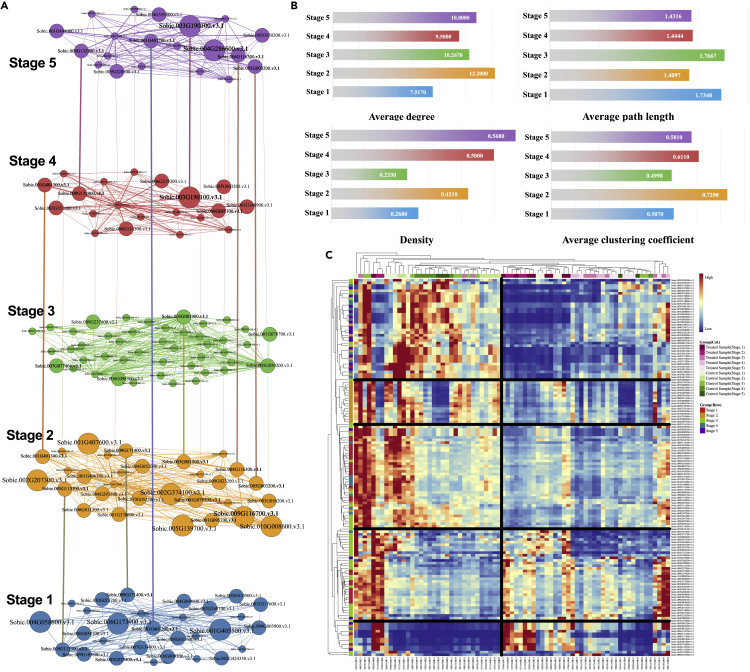
Multilayer differential co-expression network analysis for the top-20 ranked genes at the five stages (A) The constructed multilayer network for the selected genes. The inter layer edges connect the same gene in two layers; node sizes are proportional to their evidence of drought responsiveness (whether they are reported to be drought responsive (Table S1) or are annotated with drought-related GO biological processes. The largest nodes are both reported in existing references and functionally annotated). (B) Density, average degree, average path length, and average clustering coefficient for the network at each layer. (C) Clustering analysis of the expression profiles for the top-20 ranked genes in all samples. The clustering analysis is based on normalized data, which is performed by the average linkage method and Euclidean distance.

Hereinafter, based on the identified top-20 ranked genes, we construct a temporal multilayer differential co-expression network to explore the selected genes (Figure 4A). The multilayer network is constructed as follows. Firstly, we extract subnetworks of the WGDCNs for the top-20 ranked genes at each stage. The subnetwork for each stage serves as one layer. The weights of intralayer edges are the same as those in the WGDCNs. Secondly, interlayer edges are added, which connect the same gene at two different layers. Structural analysis reveals that the temporal network at Stage 2 encompasses the largest average degree and average clustering coefficient, and it has the lowest average path length, which indicates that the associated network has small-world property (Figure 4B). The subnetworks at Stages 4 and 5 are more densely connected than the other stages, which reveal that relatively more rewiring events among the selected genes have been triggered by drought stress at the reproductive growth stages. The expression profiles of the identified genes show some patterns in samples under treatment and control (Figure 4C).

To evaluate the overlap of nodes across layers, we compute the Jaccard similarity coefficient (Wang and Wang, 2022) according to

$$Overlap\left(i,j\right)=\frac{\left|A_{i}\cap A_{j}\right|}{\left|A_{i}\cup A_{j}\right|},i,j=1,2,3,4,5.$$

Here, $A_{i}$ denotes the selected gene set at the *i*’th stage (i=1,2,3,4,5). For the five-layer network, we obtain

$$Overlap=\left(\begin{array}{lllll} 1 & 0.0339 & 0 & 0 & 0.0204\\ 0.0339 & 1 & 0.0133 & 0.0408 & 0.0400\\ 0 & 0.0133 & 1 & 0 & 0\\ 0 & 0.0408 & 0 & 1 & 0.1282\\ 0.0204 & 0.0400 & 0 & 0.1282 & 1 \end{array}\right).$$

The overlaps between different layers are very low, which may reveal that there are considerable differences on rewiring patterns among different developmental stages of *Sorghum*. Especially, the overlaps among the first three stages are quite low, which may be due to the fact that the first three stages are developmental growth stages, quickly growth of the plants leads to great phenotypical differences, as well as great differences on the associated crucial genes. However, the overlap between Stage 4 and Stage 5 reaches 0.1282, which suggests that the two stages share comparably more common genes than those in the first three stages. Several common genes in the last two stages continuously play functional roles under drought stress. Stage 3 shares the least common genes with the other stages, which well separates the pre-flowering period (Stages 1 and 2) and the post-flowering period (Stages 4 and 5). Actually, there are 57 crucial genes in the pre-flowering period and 31 crucial genes in the post-flowering period, which may indicate that drought responsiveness in *Sorghum* is more complex before flowering. Moreover, the genes screened at Stage 2 have overlaps with the other four stages, indicating that Stage 2 may be a very important developmental stage, which closely relate to the whole life of *Sorghum*. In the face of drought environment, we should pay special attention on the prevention of pre-flowering drought and enhance defensive measures at Stage 2 to reduce the influence of abiotic stress on crops.

During the growth of *Sorghum*, some genes exert drought responsiveness in multiple periods, which may play roles in continuously alleviating the impact of external drought stress. For example, Sobic 0.003G081900.v3.1 is upregulated in both Stage 2 ($log_{2}\left(FC\right)=7.5911$) and Stage 3 ($log_{2}\left(FC\right)=3.4221$); Sobic.010G17800.v3.1 is downregulated at both Stage 3 ($log_{2}\left(FC\right)=-1.1637$) and Stage 4 ($log_{2}\left(FC\right)=-1.7968$); Sobic.010G1778002v3.1 is downregulated at Stages 2, 4, and 5. Though Sobic.003G190100.v3.1 is not a DEG, it is homologous with ALPHAVPE, and is identified as crucial genes both at Stages 4 and 5, which can trigger considerable differential co-expression relationships with the other genes. In general, multilayer network analysis reveals that the crucial genes can trigger extensive temporal co-expression changes under drought stress, and there are certain correlations among different stages.

### Crucial gene regulatory module for drought responsiveness in *Sorghum*

**Figure 5:**
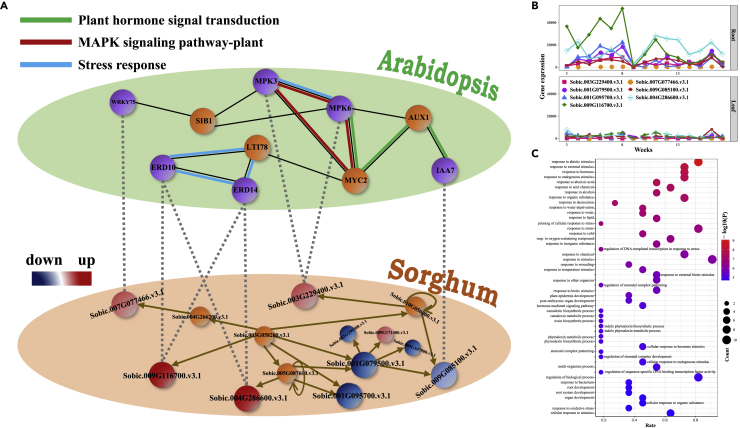
Crucial gene regulatory module for drought responsiveness in *Sorghum* (A) A two-layer network for crucial genes in *Sorghum* and their homologous in *Arabidopsis*. The lower layer represents the gene regulatory network for crucial genes in *Sorghum* (Figure S3). Upregulated genes are shown in red; while downregulated genes are shown in blue. The upper layer represents the PPI network for some homologous genes in *Arabidopsis* (Based on STRING). Interlayer links represent the homologous relationships between genes in *Arabidopsis* and *Sorghum*. (B) Changes of gene expression levels in roots and leaves for some crucial genes. The *Y* axis represents the absolute differences of gene expression between treated samples and control samples. (C) The enriched top-50 GO biological processes (with the smallest P values) for genes in the upper layer of panel A. The *X* axis represents the proportion of genes involving in a biological process.

The multilayer GDCN only reflects differential co-expression patterns among genes; based on PlantRegMap, we can further predict the possible gene interactions and explore the gene regulatory module for drought responsiveness in *Sorghum* (Figures S2 and S3). For the selected top-20 ranked genes, PlantRegMap predicts that Sobic.003G229400.v3.1, Sobic.009G116700.v3.1, Sobic.001G079500.v3.1, Sobic.001G095700.v3.1, Sobic.007G077466.v3.1, Sobic.009G085100.v3.1, and Sobic.004G286600.v3.1 have regulation relationships (Figures 5A and S2). Moreover, the expression of these genes changed more severely in roots than in leaves (Figure 5B). Further based on the STRING database, the homologous genes in *Arabidopsis* are also connected in the protein-protein interaction (PPI) network (Figures 5A and S3). The PPI network consists of several famous genes that have been reported to be closely related to drought stress, such as MPK6, MYC2, IAA7, ERD14, ERD10, WRKY75, and AUX1. The associated genes in the PPI network involve in plant hormone signal transduction, MAPK signaling pathway, and stress response. Enrichment analysis shows that genes in the homologous network are enriched in various stress-responsive processes, including response to desiccation, water deprivation, and water (Figure 5C).

The homologous genes in Figure 5A not only involve in drought-related biological processes but also relate to hormone-related (including abscisic acid (ABA) and jasmonic acid) biological processes and MAPK cascades (Figure 5C). Actually, phytohormones play an important role in regulating drought stress. Plants can sense and respond environmental changes via a series of hormone-mediated signal cascades. ABA is a common hormone in plants. It not only plays a key role during the growth and development of plants but also closely relates to drought. Actually, many genes in plants are regulated by both ABA-dependent and ABA-independent pathways to respond to drought (Riyazuddin et al., 2022; Yao et al., 2021), such as dehydrin (DHN) genes. In fact, Sobic.009G116700.v3.1 and Sobic.004G286600.v3.1 are possibly homologous with ERD10 and ERD14 in the DHN family. The DHN proteins are highly hydrophilic and perform multifaceted roles in the protection of plant cells under drought stress. For ABA-dependent pathways, the signal of drought stress is perceived by different receptors which may lead to an accumulation of ABA and decreased contents of other plant hormones. The activated hormonal signaling cascade may trigger the expression of different DHN genes that participate in drought stress tolerance by inhibiting the ROS accumulation and lipid peroxidation and protecting the photosynthetic machinery (Riyazuddin et al., 2022). For ABA-independent pathways, it is reported that fully intrinsically disordered DHN ERD14 protein might protect and even activate redox enzymes through the direct effect on the activity of glutathione transferase PHI9 in *Arabidopsis*, and thus help plants to survive oxidative stress under drought stress (Nguyen et al., 2020). At the same time, MAPK cascades are an important signaling module in responding to drought. It is demonstrated that the MAPK pathway involves in mRNA decapping via MPK6-DCP1-DCP5 pathway, playing a role in dehydration stress response (Xu and Chua, 2014). Sobic.003G229400.v3.1 is highly homologous with MPK3 and MPK6, which indicates the role of Sobic.003G229400.v3.1 under drought stress in *Sorghum*.

In addition to the hormone signal transduction pathways and the MAPK cascades, the WRKY TF family also plays an important role in responding to various abiotic stresses. Sobic.007G077466.v3.1 is homologous with WRKY75, which is defined as a crucial gene at Stage 3. It is reported that WRKY75 can participate in regulating gibberellin-mediated flowering time through the interaction with DELLAs, and it involves in the growth of roots. It is also reported that PtrWRKY75 acts on the upstream of PAL1 and directly regulates the expression of PAL1 by binding to the promoter of PAL1, and the activated PAL1 increases the accumulation of ROS by promoting the biosynthesis of salicylic acid, which eventually leads to the size of stomatal pore narrowing, thereby enhancing the drought resistance of plants (Zhang et al., 2020). Moreover, Sobic.001G079500.v3.1, Sobic.001G095700.v3.1, Sobic.004G286600.v3.1, and Sobic.009G116700.v3.1 take part in responding to water deprivation; these genes are directly or indirectly regulated by Sobic.003G058200.v3.1. Sobic.003G229400.v3.1, Sobic.007G077466.v3.1, and Sobic.009G085100.v3.1 are also regulated by Sobic.003G058200.v3.1.

In summary, a drought-responsive gene regulatory module for *Sorghum* is established, which involves in plant hormone signal transduction, MAPK cascades, and transcriptional regulation. Interestingly, Sobic.003G058200.v3.1, Sobic.005G087600.v3.1, Sobic.001G079500.v3.1, Sobic.001G095700.v3.1, Sobic.010G056000.v3.1, and Sobic.009G085100.v3.1 consist of several feedforward loops (FFLs) (Mangan and Alon, 2003; Goentoro et al., 2009; Wang et al., 2012). It is reported that FFLs can be served as either a sign-sensitive delay element (coherent FFLs) or a pulse generator and response accelerator (incoherent FFLs) (Mangan and Alon, 2003). Incoherent FFLs may be also served as a fold-change detector (Goentoro et al., 2009). Superior functions of the FFLs can well regulate the sensitivity of plant to sense and respond to drought stress.

## Discussion

With global warming and the intensifying contradiction between water supply and demand, drought has become the most important abiotic factor affecting food production in the world. However, drought-responsive mechanisms of crops are still largely unknown. *Sorghum* is a typical crop with strong drought resistance, which is an ideal crop to explore drought-responsive mechanisms. The investigations of *Sorghum* are of great significance in cultivating novel drought-resistant varieties, and in promoting sustainable agricultural development.

In this paper, to explore drought-responsive crucial genes from RNA-seq data of *Sorghum*, we establish rigorous statistical procedures. Firstly, in order to exclude redundant genes and reduce the subsequent computational burden, the MV test is performed on samples at each stage; genes that show certain dependence with the treatments are retained as candidate genes for subsequent analysis. Secondly, based on the GCC, we construct WGDCN for candidate genes at each stage. It is reported that the GCC is more robust against data processing, and it is appropriate to evaluate nonlinear relationships under small sample sizes (Wang and Wang, 2022). Finally, the WGDCN and the HMRF model are combined to calculate the posterior probabilities of candidate genes. GO enrichment analysis reveals that the identified top-20 genes are enriched in drought-related biological processes. Gene sequence alignment analysis reveals that some genes are highly homologous with drought-related genes in *Arabidopsis*. Multilayer differential co-expression network analysis shows that considerable crucial genes can trigger differential co-expression patterns at different stages. Further based on the PPI network in *Arabidopsis* and the predicted gene interactions in *Sorghum*, a possible drought-responsive module in *Sorghum* is established and discussed.

Except the proposed method, there are many other methods to explore the data in this paper. For example, we recently propose an algorithm to construct gene differential co-expression network, and based on the GDCN and the traditional degree, closeness, and betweenness centralities, crucial genes that may be associated with drought stress can be also explored (Bi and Wang, 2022). Comparing the results from the HMRF-based method and the GDCN-based method, 4,5,5,18,16 common crucial genes are selected in the top-20 ranking lists at the five stages, respectively (Table 2), which demonstrates the consistence of the proposed method with the existing ones. More importantly, several different potentially critical genes are screened by the proposed HMRF-based method, including Sobic.003G229400.v3.1, Sobic.009G116700.v3.1, Sobic.001G095700.v3.1, Sobic.007G077466.v3.1, and Sobic.009G085100.v3.1. The additionally selected genes are demonstrated to be more likely to play a key role in drought responsiveness of *Sorghum* (see Figure 5), which further reveals the merit of the proposed method.

**Table 2:** Commonly selected top-20 ranked crucial genes according to the proposed HMRF-based method and the GDCN-based method

Stage 1

Sobic.005G113300.v3.1, Sobic.006G108400.v3.1, Sobic.009G005900.v3.1, Sobic.009G171400.v3.1.

**Stage 2**

Sobic.001G079500.v3.1, Sobic.001G370600.v3.1, Sobic.001G406300.v3.1, Sobic.002G374100.v3.1, Sobic.003G376700.v3.1.

**Stage 3**

Sobic.005G050200.v3.1, Sobic.010G178000.v3.1, Sobic.003G081900.v3.1, Sobic.003G323500.v3.1, Sobic.006G276700.v3.1.

**Stage 4**

Sobic.007G092900.v3.1, Sobic.003G190100.v3.1, Sobic.004G247000.v3.1, Sobic.010G177800.v3.1, Sobic.001G148900.v3.1, Sobic.007G093000.v3.1, Sobic.007G047300.v3.1, Sobic.001G401300.v3.1, Sobic.009G132900.v3.1, Sobic.006G219300.v3.1, Sobic.010G178000.v3.1, Sobic.008G114300.v3.1, Sobic.001G351000.v3.1, Sobic.005G107900.v3.1, Sobic.003G151600.v3.1, Sobic.005G003200.v3.1, Sobic.007G058800.v3.1, Sobic.001G291300.v3.1.

**Stage 5**

Sobic.004G159733.v3.1, Sobic.007G092900.v3.1, Sobic.010G178000.v3.1, Sobic.007G093000.v3.1, Sobic.009G132900.v3.1, Sobic.005G003200.v3.1, Sobic.007G058800.v3.1, Sobic.003G190100.v3.1, Sobic.005G050200.v3.1, Sobic.004G286600.v3.1, Sobic.005G126200.v3.1, Sobic.010G273800.v3.1, Sobic.001G498000.v3.1, Sobic.004G339800.v3.1, Sobic.009G228100.v3.1, Sobic.006G026700.v3.1.

It is noted that some of the findings in this paper coincide with existing works (Paterson et al., 2009). It is reported that drought responsiveness in *Sorghum* involves many biological processes (Paterson et al., 2009), including the response to salicylic acid, response to jasmonic acid, defense response, response to fungus, and regulation of defense response. Enrichment analysis in this paper shows that the identified crucial genes are enriched in these biological processes. It is also reported that DEGs for pre-flowering stages are more than those for post-flowering stages, and changes of gene expression in pre-flowering stages are far more complex (Paterson et al., 2009). However, in this paper, the amount of the identified crucial genes for pre-flowering stages are more than those for post-flowering stages, and the overlaps of the top-20 ranked genes among the three pre-flowering stages are quite low, which coincide with the existing work. These results further support the effectiveness of the findings in this paper.

There are several advantages of this study. Firstly, different from existing methods (Hou et al., 2014), the proposed method only relies on RNA-seq data, which is appropriate for the cases without GWAS signals. Secondly, the resolution of the proposed method is higher than the MV test, which indicates that the HMRF can more precisely distinguish the crucialness of genes in responding to drought stress in *Sorghum*. Thirdly, the GCC-based approach of WGDCN is appropriate for cases with small sample sizes, which overcomes the deficiency of the traditional PCC- or SCC-based methods. Fourthly, the associated investigations consider different developmental stages of *Sorghum*; crucial genes are analyzed via temporal multilayer differential co-expression network and predicted gene interaction network; a crucial gene regulatory module is established, which regulates drought responsiveness via plant hormone signal transduction, MAPK cascades, and transcriptional regulations.

This paper only explores crucial drought-responsive genes in the root parts of *Sorghum*; it is interesting to further consider the data from the leaf parts. Moreover, based on the time series data of *Sorghum*, it is possible to construct multilayer co-expression network and to further explore useful bioinformatics. It is also interesting to establish some methods based on time series analysis to further explore the considered data. It is also noted that the proposed method can be used to explore other omics data for various organisms. All of the mentioned issues will be our future research directions. As a summary, the associated investigations not only provide rigorous theoretical foundations for exploring crucial phenotype-related genes from RNA-seq data but also provide promising target genes for molecular breeding of improved *Sorghum* varieties.

### Limitations of the study

There are some limitations in the current investigation. Firstly, the setting of hyperparameters in the HMRF needs to be further improved. For simplicity, we set the hyperparameters of the posterior probabilities of nodes as $\tau_{1}=\tau_{2}=0.01$ (Method details), which actually assumes that the contribution of two genes that are both associated with drought stress is the same as that they are both un-associated. Another parameter *h* is determined by the 90% quantile of the potentially associated state, which mainly considers the parameter settings in previous research (Hou et al., 2014) and the characteristics of actual data. Secondly, since GO annotations of genes in *Sorghum* are still largely incomplete (Paterson et al., 2009), the functions of some of the identified genes are unknown. Some detailed biological experimental validations of the selected crucial genes need to be further performed. Thirdly, the proposed method relies on several hard cutoff thresholds. The hard cutoff thresholds for GCC determine the densities of the constructed WGDCN; the P value from the MV test determines the retained candidate genes. The selection of the cutoff thresholds mainly considers the balance between computational burden and information loss. Finally, the samples are manually divided into five developmental stages, which make the amounts of samples at different stages comparable and consider the phenotype features of *Sorghum*. It will be an interesting topic to group samples according to some properly designed algorithms for optimal parting of ordered samples (Fisher, 1958).

## STAR★Methods

### Key resources table

**Table:** 

REAGENT or RESOURCE	SOURCE	IDENTIFIER
**Deposited data**		

RNA-seq data	NCBI	GEO: GSE128441
Source code	Github	https://github.com/98YiLin/EDCG.git

**Software and algorithms**		

R ×64 3.6.1	R Software	https://cran.r-project.org/
Gephi 0.9.5	Gephi	https://gephi.org/

### Resource availability

#### Lead contact

Further information and requests for resources should be directed to and will be fulfilled by the lead contact, Pei Wang (wp0307@126.com, wangpei@henu.edu.cn).

#### Materials availability

This study did not generate any new material.

### Experimental model and subject details

Our study does not use typical experimental models in the life sciences.

### Method details

#### RNA-seq data for Sorghum

The RNA-seq data for *Sorghum* is obtained from NCBI with accession number GSE128441, which is a part of the five-year EPICON project (Varoquaux et al., 2019). In the EPICON project, field-based, temporal transcriptomic data for two genotypes of *Sorghum* has been sequenced. The two genotypes are the pre-flowering drought-tolerant genotype RTx430 and the post-flowering tolerant variety BTx642 (Smith et al., 1985; Thomas and Howarth, 2000). Three experimental settings are considered: pre-flowering drought, post-flowering drought and normal watering. Almost 400 samples, ranging from week 3 to week 17, are sampled weekly from leaves and roots of the two genotypes. Each sample averagely detects the expression of 22066 genes.

We consider the root samples of BTx642 under pre-flowering drought, post-flowering drought and normal watering conditions. The main reasons are as follows: firstly, the BTx642 plants can stay green and perform active photosynthesis under drought stress, which demonstrate obvious drought resistance (Rosenow et al., 1983); Secondly, roots not only play an important role in absorbing water and nutrients, but also is pivotal in responding to various adverse environmental stresses, such as drought, low temperature (Takeuchi et al., 2011). When plant encounters drought, its roots can promptly sense the coercive changes and quickly make adaptive adjustments for self-growth. Additionally, an existing research reports that roots of *Sorghum* encompass more DEGs than leaves under drought stress at the seeding stage (Zhang et al., 2019). In the following, in order to comprehensively explore crucial genes that respond to drought stress at different developmental stages, we combine pre-flowering (from week 3 to week 8) and post-flowering (from week 9 to week 17) drought stresses as treatments. By considering the growth characteristics of graminaceous crops and the number of samples, the whole growth period of *Sorghum* is manually divided into five stages, with each three weeks as a developmental stage.

Mathematically, we denote the expression of *M* genes in *n* samples ($n_{t}$ treated samples and $n_{c}$ control samples, $n_{t}+n_{c}=n$) as

$$X={\left(x_{ij}\right)}_{n\times M}={\left(\begin{array}{l} X_{\left(1\right)},X_{\left(2\right)},\ldots ,X_{\left(n\right)} \end{array}\right)}^{T}=\left(\begin{array}{l} X_{1},X_{2},\ldots ,X_{M} \end{array}\right),$$

where $X_{i}={\left(x_{1i},x_{2i}\text{,}\cdots ,x_{ni}\right)}^{T}$ is the observations for the *i*’th gene (i=1,2,⋯,M) and $X_{\left(j\right)}={\left(x_{j1},x_{j2},\cdots ,x_{jM}\right)}^{T}$ represents the *j*’th sample (j=1,2,⋯,n). Meanwhile, the observations for the binary response variable Y is denoted as $Y={\left(y_{1},y_{2},\cdots ,y_{n}\right)}^{T}$. $y_{j}=1$ if the *j*’th sample is an experimentally treated sample, and otherwise $y_{j}=0\;\left(j=1,2,\cdots ,n\right)$.

#### Mean variance test

The mean variance (MV) test (Cui and Zhong, 2019) can effectively exclude redundant genes, and thus reduce the subsequent computational burden. Given a random variable X and a categorical response variable Y, the statistical hypothesis for the MV test is

$$H_{0}:F_{\theta }\left(x\right)=F\left(x\right)\text{ for any}\;x\text{ and }\theta ;\;H_{1}:F_{\theta }\left(x\right)\neq F\left(x\right)\text{ for some }x\text{ and }\theta ,\theta =1,2,\cdots ,C.$$

Here, $F_{\theta }\left(x\right)$ denotes the conditional distribution function of X given Y=θ, and F(x) denotes the distribution function of X; *C* denotes the total categories of Y, C=2 for binary response variable.

Cui et al. (Cui and Zhong, 2019) proposed a sample-level MV index (Equation 1), which can be used to test the independence between the expression profile of the *i*’th gene $X_{i}={\left(x_{1i},x_{2i}\text{,}\cdots ,x_{ni}\right)}^{T}$ and the response $Y={\left(y_{1},y_{2},\cdots ,y_{n}\right)}^{T}$,

(Equation 1) $$T_{n}^{\left(i\right)}=n\hat{MV}\left(X_{i}|Y\right)=\overset{C}{\underset{\theta =1}{\sum }}\overset{n}{\underset{j=1}{\sum }}{\hat{p}}_{\theta }{\left\{{\hat{F}}_{\theta }\left(x_{ji}\right)-\hat{F}\left(x_{ji}\right)\right\}}^{2},$$

where

$${\hat{F}}_{\theta }\left(x\right)=\frac{\sum_{v=1}^{n}I\left\{x_{vi}\leq x,y_{v}=\theta \right\}}{\sum_{v=1}^{n}I\left\{y_{v}=\theta \right\}}$$

is the empirical conditional distribution function of $X_{i}$ given response variable Y=θ, $\hat{F}\left(x\right)=n^{-1}\sum_{v=1}^{n}I\left\{x_{vi}\leq x\right\}$ is the empirical unconditional distribution function of $X_{i}$, and ${\hat{p}}_{\theta }=n^{-1}\sum_{v=1}^{n}I\left\{y_{v}=\theta \right\}$ denotes the sample proportion of the θ′th category. I(.) is an indicator function. Larger $T_{n}^{\left(i\right)}$ provides a stronger evidence against the null hypothesis $H_{0}$, indicating that the correlation between $X_{i}$ and the binary response variable Y is higher.

For small sample size, Cui et al. (Cui and Zhong, 2019) developed a permutation test to obtain the P value for the MV test. Procedures are as follows:

Step 1: Compute the MV test statistic for the given sample $\left\{\left(x_{ji}\text{,}y_{j}\right),j=1,2,\cdots ,n\right\}$ by

$$T_{n0}^{\left(i\right)}=n\hat{MV}\left(X_{i}|Y\right)=\overset{C}{\underset{\theta =1}{\sum }}\overset{n}{\underset{j=1}{\sum }}{\hat{p}}_{\theta }{\left\{{\hat{F}}_{\theta }\left(x_{ji}\right)-\hat{F}\left(x_{ji}\right)\right\}}^{2}.$$

Step 2: Generate a permuted response sample $Y^{\text{∗}}={\left(y_{1}^{\text{∗}}\text{,}y_{2}^{\text{∗}}\text{,}\cdots \text{,}y_{n}^{\text{∗}}\right)}^{T}$ from the original response vector, and compute the corresponding MV index $T_{n}^{\left(i\right)\text{∗}}=n\hat{MV}\left(X_{i}|Y^{\text{∗}}\right)$.
Step 3: Repeat Step 2 for *K* times and obtain *K* permuted MV statistics $T_{n1}^{\left(i\right)\text{∗}},T_{n2}^{\left(i\right)\text{∗}},\cdots ,T_{nK}^{\left(i\right)\text{∗}}$. The P value is estimated by

$$P_{MV}^{\left(i\right)}=\frac{1}{K}\overset{K}{\underset{k=1}{\sum }}I\left(T_{nk}^{\left(i\right)\text{∗}}\geq T_{n0}^{\left(i\right)}\right),i=1,2,\cdots ,M.$$

In this paper, for each gene, we set K=5000. If $P_{MV}^{\left(i\right)}\leq 0.01$, the null hypothesis should be rejected, we have reason to believe that there is correlation between $X_{i}$ and *Y*; Otherwise, the *i*’th gene is deemed to be independent with *Y*, and it is neglected in the subsequent analysis.

#### Weighted gene differential co-expression network

To reveal whether a gene can trigger differential co-expression patterns or rewiring between treatment and control, weighted gene differential co-expression networks (WGDCNs) will be constructed. Since Pearson correlation coefficient (PCC) (Hudson et al., 2009) and Spearman correlation coefficient (SCC) (Sedgwick, 2014) all rely on considerable samples, and they are sensitive to data processing (Wang, 2021), Gray correlation coefficient (GCC) will be used to evaluate the co-expression relationships between genes. Specifically, when the *p*’th gene is taken as a reference, the GCC between the *p*’th and the *q*’th genes can be obtained according to (Wang and Wang, 2022; Chen and Liu, 2021)

(Equation 2) $$r_{pq}=\frac{1}{n}\sum_{k=1}^{n}\frac{{\text{min}}_{s\in \left\{1\text{,}2\text{,}\cdots \text{,}m\right\}}{\text{min}}_{t\in \left\{1\text{,}2\text{,}\cdots \text{,}n\right\}}|x_{tp}-x_{ts}|+\rho {\text{max}}_{s\in \left\{1\text{,}2\text{,}\cdots \text{,}m\right\}}{\text{max}}_{t\in \left\{1\text{,}2\text{,}\cdots \text{,}n\right\}}|x_{tp}-x_{ts}|}{\left|x_{kp}-x_{kq}|+\rho {\text{max}}_{s\in \left\{1\text{,}2\text{,}\cdots \text{,}m\right\}}{\text{max}}_{t\in \left\{1\text{,}2\text{,}\cdots \text{,}n\right\}}|x_{tp}-x_{ts}\right|}.$$

Here, $r_{pq}\in \left[0\text{,}1\right],q=1,2,\cdots ,m$; *ρ* is called resolution ratio, which is usually taken as 0.5. *m* denotes the number of genes with $P_{MV}\leq 0.01$. Since the GCC relies on reference sequence, generally, $r_{pq}\neq r_{qp}$. To overcome this disadvantage, we correct the GCC between the *p*’th and the *q*’th genes as $\left(r_{pq}+r_{qp}\right)/2$. Samples under treatments and controls are separately considered, and we denote $r_{pq}^{treat}$ and $r_{pq}^{control}$ as the corrected GCC between the two genes in treated and control samples respectively.

Based on the GCC, the WGDCN for a specific developmental stage is constructed as follows. We set $r_{0}=0.9$ as a hard threshold (Such hard threshold mainly considers the density of the constructed network and information loss). If the correlation between two genes satisfied $\left(r_{pq}^{treat}-r_{0}\right)\left(r_{pq}^{control}-r_{0}\right)\leq 0$, then, genes *p* and *q* are differentially co-expressed between treatments and controls, and an undirected edge between the two genes is added. Edge weight is defined as

(Equation 3) $$rewire_{pq}=|r_{pq}^{treat}-r_{pq}^{control}|.$$

Here, $rewire_{pq}$ reflects the importance/strength of rewiring between the two genes at the given developmental stage.

#### Hidden Markov random field model

Suppose G=(υ,ε) is an undirected graph, υ={1,2,⋯,m} is the set of nodes (genes); ε is the edge set, $e_{pq}=1$ if the *p*’th and the *q*’th genes are connected, and their connection strength is $rewire_{pq}$ (Equation 3). Denote $\omega_{p}$ as the true association status of the *p*’th gene with drought stress, $\omega_{p}=+1$ if gene *p* is associated with drought stress, otherwise $\omega_{p}=-1$. For simplicity, $\omega_{p}$ is called as the label of gene *p*, and $\text{Ω}=\left\{\omega_{1}\text{,}\omega_{2}\text{,}\cdots \text{,}\omega_{m}\right\}$ is called as the label vector or a configuration for the node set υ.

Assume that neighbored genes tend to have similar association status (Chen et al., 2011; Hou et al., 2014), the probability distribution of network configuration can be described by an Ising model (Kindermann and Snell, 1980), which is defined as $P\left(\omega_{1}\text{,}\omega_{2}\text{,}\cdots \text{,}\omega_{m}\right)=$

(Equation 4) $$\frac{1}{Z}exp\left\{-h\overset{m}{\underset{p=1}{\sum }}I\left(\omega_{p}=+1\right)+\tau_{1}\underset{e_{pq}=1}{\sum }rewire_{pq}\cdot I\left(\omega_{p}=+1,\omega_{q}=+1\right)-\tau_{2}\underset{e_{pq}=1,rewire_{pq}>\delta }{\sum }rewire_{pq}\cdot I\left(\omega_{p}=-1,\omega_{q}=-1\right)\right\}.$$

Here, the partition function Z=

$$\underset{\text{Ω}}{\sum }exp\left\{-h\overset{m}{\underset{p=1}{\sum }}I\left(\omega_{p}=+1\right)+\tau_{1}\underset{e_{pq}=1}{\sum }rewire_{pq}\cdot I\left(\omega_{p}=+1,\omega_{q}=+1\right)-\tau_{2}\underset{e_{pq}=1,rewire_{pq}>\delta }{\sum }rewire_{pq}\cdot I\left(\omega_{p}=-1,\omega_{q}=-1\right)\right\};$$

I(⋅) is an indicator function; $h,\tau_{1},\tau_{2}$ are hyper-parameters. *h* is a constant, which is defined as the probability of being drought stress associated if the gene is isolated. $\tau_{1}$ represents the contributions of the rewired drought-associated gene pairs; while $\tau_{2}$ reflects the contributions of gene pairs that are not associated with drought stress (Chen et al., 2011). δ=0.95, $rewire_{pq}>\delta$ indicates the rewiring between genes *p* and *q* under treatments and controls is significant. It is noted that an underlying biological hypothesis behind model (Equation 4) is that, the co-expression difference of genes under two different experimental conditions can actually reflect their phenotype differences. That is, the model follows the guilt-by-rewiring principle (Hou et al., 2014).

Based on the formula of conditional probability, we obtain

(Equation 5) $$P\left(\omega_{p}=+1|\omega_{N_{p}}\right)=\frac{P\left(\omega_{p}=+1,\omega_{N_{p}}\right)}{P\left(\omega_{N_{p}}\right)},P\left(\omega_{p}=-1|\omega_{N_{p}}\right)=\frac{P\left(\omega_{p}=-1,\omega_{N_{p}}\right)}{P\left(\omega_{N_{p}}\right)}.$$

According to Equations 4 and 5, we have

$$\frac{P\left(\omega_{p}=+1|\omega_{N_{p}}\right)}{P\left(\omega_{p}=-1|\omega_{N_{p}}\right)}=\frac{P\left(\omega_{p}=+1,\omega_{N_{p}}\right)}{P\left(\omega_{p}=-1,\omega_{N_{p}}\right)}=\frac{exp\left\{-h+\tau_{1}\sum_{e_{pq}=1}rewire_{pq}\cdot I\left(\omega_{q}=+1\right)\right\}}{exp\left\{-\tau_{2}\sum_{e_{pq}=1,rewire_{pq}>\delta }rewire_{pq}\cdot I\left(\omega_{q}=-1\right)\right\}}=exp\left\{-h+\tau_{1}\sum_{e_{pq}=1}rewire_{pq}\cdot I\left(\omega_{q}=+1\right)+\tau_{2}\sum_{e_{pq}=1,rewire_{pq}>\delta }rewire_{pq}\cdot I\left(\omega_{q}=-1\right)\right\}.$$

Combining with $P\left(\omega_{p}=+1|\omega_{N_{p}}\right)+P\left(\omega_{p}=-1|\omega_{N_{p}}\right)=1$, Equation 5 can be further rewritten as:

$$P\left(\omega_{p}=+1|\omega_{N_{p}}\right)=\frac{exp\left(F\right)}{1+exp\left(F\right)},P\left(\omega_{p}=-1|\omega_{N_{p}}\right)=\frac{1}{1+exp\left(F\right)},$$

where $F=-h+\tau_{1}\sum_{e_{pq}=1}rewire_{pq}\cdot I\left(\omega_{q}=+1\right)+\tau_{2}\sum_{e_{pq}=1,rewire_{pq}>\delta }rewire_{pq}\cdot I\left(\omega_{q}=-1\right)$.

Furthermore, the conditional distribution of the associated status for gene *p* can be obtained as:

(Equation 6) $$logitP\left(\omega_{p}|\omega_{N_{p}}\right)=ln\frac{P\left(\omega_{p}|\omega_{N_{p}}\right)}{1-P\left(\omega_{p}|\omega_{N_{p}}\right)}=-h+\tau_{1}\underset{e_{pq}=1}{\sum }rewire_{pq}\cdot I\left(\omega_{q}=+1\right)+\tau_{2}\underset{e_{pq}=1,rewire_{pq}>\delta }{\sum }rewire_{pq}\cdot I\left(\omega_{q}=-1\right).$$

Here $N_{p}=\left\{q:\left\langle p,q\right\rangle \in \varepsilon \right\}$ denotes the neighbor set of gene *p*; $\omega_{N_{p}}$ denotes the label set of gene *p*’s neighbors; logit(P)=ln(P/(1−P)) is the logit function.

Given the joint probability of the labels for all genes, the posterior probability of network configuration can be inferred through the following Bayesian framework:

(Equation 7) P(Ω|ξ)∝f(ξ|Ω)P(Ω).

Here, $f\left(\xi |\text{Ω}\right)=\prod_{\left\{p:\;\omega_{p}=-1\right\}}f_{0}\left(\xi_{p}\right)\cdot \prod_{\left\{p\text{,}:\text{,}\omega_{p}=+1\right\}}f_{1}\left(\xi_{p}\right)$. We can also obtain that

$$P\left(\omega_{p}=+1|\xi ,\omega_{N_{p}}\right)\propto f_{1}\left(\xi_{p}\right)P\left(\omega_{p}=+1|\omega_{N_{p}}\right),P\left(\omega_{p}=-1|\xi ,\omega_{N_{p}}\right)\propto f_{0}\left(\xi_{p}\right)P\left(\omega_{p}=-1|\omega_{N_{p}}\right).$$

In Equation 7, the observed data $\xi =\left(\xi_{1}\text{,}\xi_{2},\cdots \text{,}\xi_{m}\right)$ is taken as the normalized scores that transformed from the P value of the MV test: $\xi_{p}=\Phi^{-1}\left[1-P_{MV}\left(p\right)\right]$, Φ is the cumulative distribution function of standard normal distribution. Under the null hypothesis that the gene is not associated with drought stress, the P value follows Uniform (0,1) distribution. Similar to Chen et al., 2011, if gene *p* is not associated with drought stress, we assume the density of $\xi_{p}$ is $f_{0}\left(\xi_{p}\right)∼N\left(0\text{,}1\right)$; otherwise, $f_{1}\left(\xi_{p}\right)∼N\left(\mu_{p}\text{,}\sigma_{p}^{2}\right)$, where $\mu_{p}$ is a location parameter, $\sigma_{p}^{2}$ is a scale parameter. We consider conjugate priors:

(Equation 8) $$\mu_{p}|\sigma_{p}^{2}∼N\left(\bar{\mu }\text{,}\frac{\sigma_{p}^{2}}{a}\right),\sigma_{p}^{2}∼InverseGamma\left(\frac{g}{2}\text{,}\frac{gd}{2}\right).$$

Here, $\bar{\mu },a,g,d$ are hyperparameters, and it has been proved that the settings of these parameters have no significant effect on simulation results (Chen et al., 2011). Then the hidden states can be inferred by the iterated conditional mode algorithm (Besag, 1986; Nayak et al., 2009). The status of genes are supposed to be Markovian, we have

(Equation 9) $$P\left(\omega_{p}|\omega_{-p}\right)=P\left(\omega_{p}|\omega_{N_{p}}\right),$$

where $\omega_{-p}$ denotes the status of the node set that excludes node *p*.

Then, we further obtain

$$\frac{P\left(\omega_{p}=+1|\xi ,\omega_{-p}\right)}{P\left(\omega_{p}=-1|\xi ,\omega_{-p}\right)}\propto \frac{f_{1}\left(\xi_{p}\right)P\left(\omega_{p}=+1|\omega_{-p}\right)}{f_{0}\left(\xi_{p}\right)P\left(\omega_{p}=-1|\omega_{-p}\right)}=\frac{f_{1}\left(\xi_{p}\right)P\left(\omega_{p}=+1|\omega_{N_{p}}\right)}{f_{0}\left(\xi_{p}\right)P\left(\omega_{p}=-1|\omega_{N_{p}}\right)}=\frac{f_{1}\left(\xi_{p}\right)}{f_{0}\left(\xi_{p}\right)}exp\left\{-h+\tau_{1}\underset{e_{pq}=1}{\sum }rewire_{pq}\cdot I\left(\omega_{q}=+1\right)+\tau_{2}\underset{e_{pq}=1,rewire_{pq}>\delta }{\sum }rewire_{pq}\cdot I\left(\omega_{q}=-1\right)\right\}=exp\left\{ln\;\frac{f_{1}\left(\xi_{p}\right)}{f_{0}\left(\xi_{p}\right)}-h+\tau_{1}\underset{e_{pq}=1}{\sum }rewire_{pq}\cdot I\left(\omega_{q}=+1\right)+\tau_{2}\underset{e_{pq}=1,rewire_{pq}>\delta }{\sum }rewire_{pq}\cdot I\left(\omega_{q}=-1\right)\right\}.$$

Since $P\left(\omega_{p}=+1|\xi ,\omega_{-p}\right)+P\left(\omega_{p}=-1|\xi ,\omega_{-p}\right)=1$, we have

$$P\left(\omega_{p}=+1|\xi ,\omega_{-p}\right)=\frac{exp\left(Q\right)}{1+exp\left(Q\right)},P\left(\omega_{p}=-1|\xi ,\omega_{-p}\right)=\frac{1}{1+exp\left(Q\right)},$$

where $Q=ln\left(\frac{f_{1}\left(\xi_{p}\right)}{f_{0}\left(\xi_{p}\right)}\right)-h+\tau_{1}\sum_{e_{pq}=1}rewire_{pq}\cdot I\left(\omega_{q}=+1\right)+\tau_{2}\sum_{e_{pq}=1,rewire_{pq}>\delta }rewire_{pq}\cdot I\left(\omega_{q}=-1\right).$ Thus, the posterior distribution of the association status for gene *p* can be inferred by

(Equation 10) $$logitP\left(\omega_{p}|\xi ,\omega_{-p}\right)=ln\;\frac{P\left(\omega_{p}|\xi ,\omega_{-p}\right)}{1-P\left(\omega_{p}|\xi ,\omega_{-p}\right)}=ln\left(\frac{f_{1}\left(\xi_{p}\right)}{f_{0}\left(\xi_{p}\right)}\right)-h+\tau_{1}\sum_{e_{pq}=1}rewire_{pq}\cdot I\left(\omega_{q}=+1\right)+\tau_{2}\sum_{e_{pq}=1,rewire_{pq}>\delta }rewire_{pq}\cdot I\left(\omega_{q}=-1\right).$$

Set the initial values of parameters as $\tau_{1}=\tau_{2}=0.01$. Based on the MV test, we assign labels for genes with $P_{MV}\leq 0.005$ as “+1” (associated), and the others ($0.005<P_{MV}\leq 0.01$) as “-1”. Then, for each gene, an association potential can be obtained as

(Equation 11) $$Potential\left(p\right)=\tau_{1}\sum_{\omega_{q}=1,e_{pq}=1}rewire_{pq}+\tau_{2}\underset{\omega_{q}=-1,e_{pq}=1,rewire_{pq}>\delta }{\sum }rewire_{pq},p=1,2,\cdots ,m.$$

Parameter *h* is taken as the 90′th quantile of the potential vector, which reflects the belief that genes with marginal P value could also be related to drought stress. The iterated conditional model algorithm (Besag, 1986, Nayak et al., 2009) is further used to update these parameters. When Equation 10 converges to its local maximum (Besag, 1986), we obtain the final label of each gene and the corresponding posterior probability. Finally, genes with high posterior probabilities are selected as crucial drought responsive candidates.

#### Differentially expressed genes and GO enrichment analysis

Differential expression analysis and GO enrichment analysis are performed by OmicStudio tools (www.omicstudio.cn/tool). Genes with significant expression differences between treatments and controls are deemed as differentially expressed genes (DEGs). Mathematically, DEGs are defined as genes with $|log_{2}\left(FC\right)|>1$ and P<0.05. Here, FC denotes the fold change value between the average expression value under treatments and that under controls. In this paper, GO biological processes with P<0.1 are considered.

### Quantification and statistical analysis

All data are analyzed using R (http://www.R-project.org/, version 3.6.1) and Gephi (https://gephi.org/, version 0.9.5). Statistical tests for each analysis can be found in each figure or the main text. Here, DEGs are defined as genes with $|log_{2}\left(FC\right)|>1$ and P<0.05 (also see method details and Figure 2A). In the mean variance test, genes with $P_{MV}^{\left(i\right)}\leq 0.01$ are retained as candidate genes (also see method details).

## Data Availability

• This paper analyzes existing, publicly available data. The data can be freely downloaded from NCBI with accession number GSE128441. See method details for details. • The codes for constructing weighted gene differential co-expression network and for computing the posterior probabilities are deposited in GitHub: https://github.com/98YiLin/EDCG.git. • Any additional information required to reanalyze the data reported in this paper is available from the lead contact upon request.

## References

[bib1] Babita M., Maheswari M., Rao L.M., Shanker A.K., Rao D.G. (2010). Osmotic adjustment, drought tolerance and yield in *Castor* (*Ricinus communis* L.) hybrids. Environ. Exp. Bot..

[bib2] Besag J. (1986). On the statistical-analysis of dirty pictures. J. Roy. Stat. Soc. B.

[bib3] Bhargava S., Sawant K. (2012). Drought stress adaptation: metabolic adjustment and regulation of gene expression. Plant Breed..

[bib4] Bi Y., Wang P. (2022). Proc. 41st Chinese Control Confer., July 25-27.

[bib5] Capell T., Bassie L., Christou P. (2004). Modulation of the polyamine biosynthetic pathway in transgenic rice confers tolerance to drought stress. Proc. Natl. Acad. Sci. USA.

[bib6] Chen M., Cho J., Zhao H. (2011). Incorporating biological pathways via a Markov random field model in genome-wide association studies. PLoS Genet..

[bib7] Chen J., Bardes E.E., Aronow B.J., Jegga A.G. (2009). ToppGene suite for gene list enrichment analysis and candidate gene prioritization. Nucleic Acids Res..

[bib8] Chen G., Liu Z.P. (2021). Int. Conf. Intelligent Comput..

[bib9] Council N. (1996).

[bib10] Csermely P., Korcsmáros T., Kiss H.J.M., London G., Nussinov R. (2013). Structure and dynamics of molecular networks: a noval paradigm of drug discovery: a comprehensive review. Pharmacol. Ther..

[bib11] Cui H., Zhong W. (2019). A distribution-free test of independence based on mean variance index. Comput. Stat. Data Anal..

[bib12] Dahiya R.C., Gurland J. (1972). Pearson chi-squared test of fit with random intervals. Biometrika.

[bib13] Ding H., Yang Y., Xue Y., Seninge L., Gong H., Safavi R., Califano A., Stuart J.M. (2020). Prioritizing transcriptional factors in gene regulatory networks with PageRank. iScience.

[bib14] Fahad S., Bajwa A.A., Nazir U., Anjum S.A., Farooq A., Zohaib A., Sadia S., Nasim W., Adkins S., Saud S. (2017). Crop production under drought and heat stress: plant responses and management options. Front. Plant Sci..

[bib15] Fisher W.D. (1958). On grouping for maximum homogeneity. J. Am. Stat. Assoc..

[bib16] Flowers T.J., Yeo A.R. (1986). Ion relations of plants under drought and salinity. Funct. Plant Biol..

[bib17] Goentoro L., Shoval O., Kirschner M.W., Alon U. (2009). The incoherent feedforward loop can provide fold-change detection in gene regulation. Mol. Cell.

[bib18] Hahn L.W., Ritchie M.D., Moore J.H. (2003). Multifactor dimensionality reduction software for detecting gene-gene and gene-environment interactions. Bioinformatics.

[bib19] Hou L., Chen M., Zhang C.K., Cho J., Zhao H. (2014). Guilt by rewiring: gene prioritization through network rewiring in genome wide association studies. Hum. Mol. Genet..

[bib20] House L.R. (1985).

[bib21] Hudson N.J., Reverter A., Dalrymple B.P. (2009). A differential wiring analysis of expression data correctly identifies the gene containing the causal mutation. PLoS Comput. Biol..

[bib22] Ilhan S., Ozdemir F., Bor M. (2015). Contribution of trehalose biosynthetic pathway to drought stress tolerance of Capparis ovata Desf. Plant Biol..

[bib23] Jaiswal S.K., Mahajan S., Chakraborty A., Kumar S., Sharma V.K. (2021). The genome sequence of *Aloe vera* reveals adaptive evolution of drought tolerance mechanisms. iScience.

[bib24] Jeong H., Mason S.P., Barabási A.L., Oltvai Z.N. (2001). Lethality and centrality in protein networks. Nature.

[bib25] Johnson M., Zaretskaya I., Raytselis Y., Merezhuk Y., McGinnis S., Madden T.L. (2008). NCBI BLAST: a better web interface. Nucleic Acids Res..

[bib26] Kawata K., Hatano A., Yugi K., Kubota H., Sano T., Fujii M., Tomizawa Y., Kokaji T., Tanaka K.Y., Uda S. (2018). Trans-omic analysis reveals selective responses to Induced and basal insulin across signaling, transcriptional, and metabolic networks. iScience.

[bib27] Kindermann R., Snell J.L. (1980).

[bib28] Lü J., Wang P. (2020).

[bib29] Lü L., Chen D., Ren X.L., Zhang Q.M., Zhang Y.C., Zhou T. (2016). Vital nodes identification in complex networks. Phys. Rep..

[bib30] Lü L., Medo M., Yeung C.H., Zhang Y.C., Zhang Z.K., Zhou T. (2012). Recommender systems. Phys. Rep..

[bib31] Lee I., Blom U.M., Wang P.I., Shim J.E., Marcotte E.M. (2011). Prioritizing candidate disease genes by network-based boosting of genome-wide association data. Genome Res..

[bib32] Lilliefors H.W. (1967). On the Kolmogorov-Smirnov test for normality with mean and variance unknown. J. Am. Stat. Assoc..

[bib33] Liu R., Wang X., Aihara K., Chen L. (2014). Early diagnosis of complex diseases by molecular biomarkers, network biomarkers, and dynamical network biomarkers. Med. Res. Rev..

[bib34] Liu X., Liu Z.P., Zhao X.M., Chen L. (2012). Identifying disease genes and module biomarkers by differential interactions. J. Am. Med. Inf. Assoc..

[bib35] Ma H., Chen J., Zhang Z., Ma L., Yang Z., Zhang Q., Li X., Xiao J., Wang S. (2017). MAPK kinase 10.2 promotes disease resistance and drought tolerance by activating different MAPKs in *rice*. Plant J..

[bib36] Macciotta N.P.P., Gaspa G., Steri R., Pieramati C., Carnier P., Dimauro C. (2009). Pre-selection of most significant SNPS for the estimation of genomic breeding values. BMC Proc..

[bib37] Mace E.S., Tai S., Gilding E.K., Li Y., Prentis P.J., Bian L., Campbell B.C., Hu W., Innes D.J., Han X. (2013). Whole-genome sequencing reveals untapped genetic potential in Africa’s indigenous cereal crop *Sorghum*. Nat. Commun..

[bib38] Mangan S., Alon U. (2003). Structure and function of the feed-forward loop network motif. Proc. Natl. Acad. Sci. USA.

[bib39] Moore J.H. (2004). Computational analysis of gene-gene interactions using multifactor dimensionality reduction. Expert Rev. Mol. Diagn..

[bib40] Nayak S., Sarkar S., Loeding B. (2009). 2009 IEEE Conf. Computer Vision Patt. Recog..

[bib41] Ngara R., Goche T., Swanevelder D.Z.H., Chivasa S. (2021). *Sorghum*’s whole-plant transcriptome and proteome responses to drought stress: a review. Life.

[bib42] Nguyen P.N., Tossounian M.A., Kovacs D.S., Thu T.T., Stijlemans B., Vertommen D., Pauwels J., Gevaert K., Angenon G., Messens J., Tompa P. (2020). Dehydrin ERD14 activates glutathione transferase Phi9 in *Arabidopsis thaliana* under osmotic stress. Biochim. Biophys. Acta Gen. Subj..

[bib43] Paterson A.H., Bowers J.E., Bruggmann R., Dubchak I., Grimwood J., Gundlach H., Haberer G., Hellsten U., Mitros T., Poliakov A. (2009). The *Sorghum bicolor* genome and the diversification of grasses. Nature.

[bib44] Pezeshki S.R., Chambers J.L. (1986). Stomatal and photosynthetic response of drought-stressed cherrybark oak (*Quercusfalcata var. pagodaefolia*) and sweet gum (*Liquidambar styraciflua*). Can. J. For. Res..

[bib45] Pinheiro C., Chaves M.M. (2011). Photosynthesis and drought: can we make metabolic connections from available data?. J. Exp. Bot..

[bib46] Riyazuddin R., Nisha N., Singh K., Verma R., Gupta R. (2022). Involvement of dehydrin proteins in mitigating the negative effects of drought stress in plants. Plant Cell Rep..

[bib47] Rosenow D.T., Quisenberry J.E., Wendt C.W., Clark L.E. (1983). Drought tolerant *Sorghum* and *cotton* germplasm. Agric. Water Manag..

[bib48] Sedgwick P. (2014). Spearman’s rank correlation coefficient. Br. Med. J..

[bib49] Shang H., Liu Z.P. (2021). Prioritizing type 2 diabetes genes by weighted PageRank on bilayer heterogeneous networks. IEEE ACM Trans. Comput. Biol. Bioinf..

[bib50] Smith R.H., Bhaskaran S., Miller F.R. (1985). Screening for drought tolerance in *Sorghum* using cell culture. In Vitro Cell. Dev. Biol..

[bib51] Takeuchi K., Gyohda A., Tominaga M., Kawakatsu M., Hatakeyama A., Ishii N., Shimaya K., Nishimura T., Riemann M., Nick P. (2011). RSOsPR10 expression in response to environmental stresses is regulated antagonistically by jasmonate/ethylene and salicylic acid signaling pathways in *rice* roots. Plant Cell Physiol..

[bib52] Thomas H., Howarth C.J. (2000). Five ways to stay green. J. Exp. Bot..

[bib53] Tsugama D., Liu S., Takano T. (2012). Drought-induced activation and rehydration-induced inactivation of MPK6 in *Arabidopsis*. Biochem. Biophys. Res. Commun..

[bib54] Vanderlip R.L., Reeves H.E. (1972). Growth stages of *Sorghum* [*Sorghum bicolor*, (L.) moench.]. Agron. J..

[bib55] Vanunu O., Magger O., Ruppin E., Shlomi T., Sharan R. (2010). Associating genes and protein complexes with disease via network propagation. PLoS Comput. Biol..

[bib56] Varoquaux N., Cole B., Gao C., Pierroz G., Baker C.R., Patel D., Madera M., Jeffers T., Hollingsworth J., Sievert J. (2019). Transcriptomic analysis of field-droughted *Sorghum* from seedling to maturity reveals biotic and metabolic responses. Proc. Natl. Acad. Sci. USA.

[bib57] Wang P., Yang C., Chen H., Song C., Zhang X., Wang D. (2017). Transcriptomic basis for drought-resistance in *Brassica napus* L. Sci. Rep..

[bib61] Wang P., Wang D. (2022). Gene differential co-expression networks based on RNA-seq: construction and its applications. IEEE ACM Trans. Comput. Biol. Bioinf..

[bib58] Wang P., Yang C., Chen H., Luo L., Leng Q., Li S., Han Z., Li X., Song C., Zhang X., Wang D. (2018). Exploring transcription factors reveals crucial members and regulatory networks involved in different abiotic stresses in *Brassica napus* L. BMC Plant Biol..

[bib59] Wang Z., Yang C., Chen H., Wang P., Wang P., Song C., Zhang X., Wang D. (2018). Multi-gene co-expression can improve comprehensive resistance to multiple abiotic stresses in *Brassica napus* L. Plant Sci..

[bib60] Wang P. (2021). Statistical identification of important nodes in biological systems. J. Syst. Sci. Complex..

[bib71] Wang P., Chen S., Yang S. (2022). Recent advances on penalized regression models for biological data. Mathematics.

[bib62] Wang P., Lü J., Ogorzalek M.J. (2012). Global relative parameter sensitivities of the feed-forward loops in genetic networks. Neurocomputing.

[bib63] Wu X., Jiang R., Zhang M.Q., Li S. (2008). Network-based global inference of human disease genes. Mol. Syst. Biol..

[bib64] Xu M., Fralick D., Zheng J.Z., Wang B., Tu X.M., Feng C. (2017). The differences and similarities between two-sample T-test and paired T-test. Shanghai Arch. Psychiatry.

[bib65] Xu J., Li Y. (2006). Discovering disease-genes by topological features in human protein-protein interaction network. Bioinformatics.

[bib66] Xu J., Chua N.H. (2014). Dehydration stress activates *Arabidopsis* MPK6 to signal DCP1 phosphorylation. EMBO J..

[bib67] Yang P., Li X., Wu M., Kwoh C.K., Ng S.K. (2011). Inferring gene-phenotype associations via global protein complex network propagation. PLoS One.

[bib68] Yao T., Zhang J., Xie M., Yuan G., Tschaplinski T.J., Muchero W., Chen J.G. (2021). Transcriptional regulation of drought response in *Arabidopsis* and woody plants. Front. Plant Sci..

[bib69] Zhang D.F., Zeng T.R., Liu X.Y., Gao C.X., Li Y.X., Li C.H., Song Y.C., Shi Y.S., Wang T.Y., Li Y. (2019). Transcriptomic profiling of *Sorghum* leaves and roots responsive to drought stress at the seedling stage. J. Integr. Agric..

[bib70] Zhang Y., Zhou Y., Zhang D., Tang X., Li Z., Shen C., Han X., Deng W., Yin W., Xia X. (2020). PtrWRKY75 overexpression reduces stomatal aperture and improves drought tolerance by salicylic acid- induced reactive oxygen species accumulation in *Poplar*. Environ. Exp. Bot..

