# Supplementary material for: Exploring drought-responsive crucial genes in Sorghum
Source: iScience. 2022 Oct 14;25(11):105347. doi: 10.1016/j.isci.2022.105347 (PMC9619295; doi:10.1016/j.isci.2022.105347)
Supplement: Document S1. Figures S1–S3 and Table S1 [file mmc1.pdf]

iScience, Volume *25*

Supplemental information

Exploring drought-responsive crucial genes in *Sorghum*

Yilin Bi and Pei Wang

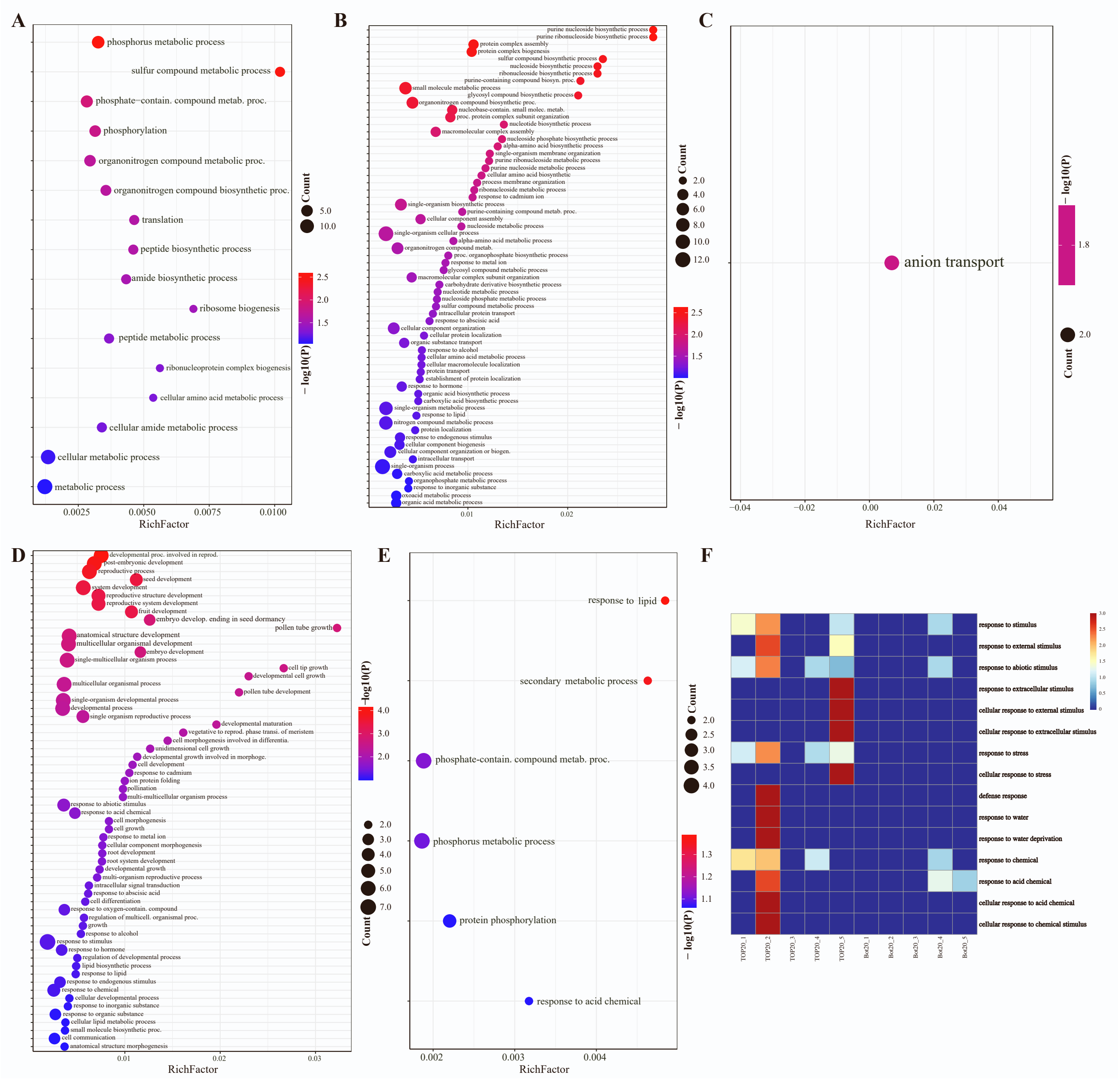

Figure S1: GO enrichment analysis for the bottom-20 ranked genes at the five stages. A-E are for the cases at stages 1-5 respectively. Biological processes with $P < 0.1$ are considered. F. Comparison of enrichment analysis results for the top-20 and bottom-20 ranked genes, where the count of genes involved in a biological pathway is standardized. Related to Fig.3.

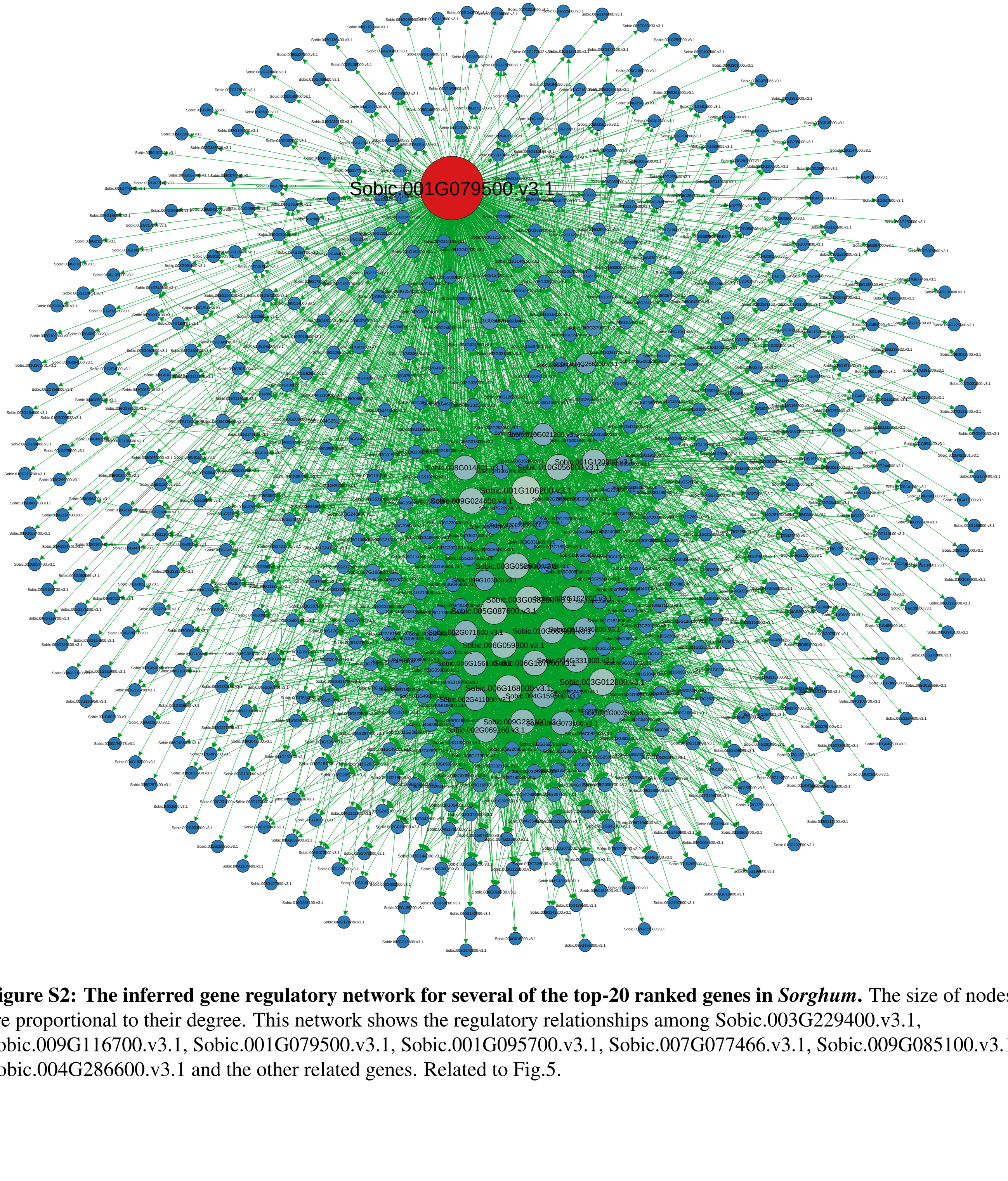

Figure S2: The inferred gene regulatory network for several of the top-20 ranked genes in *Sorghum*. The size of nodes are proportional to their degree. This network shows the regulatory relationships among Sobic.003G229400.v3.1, Sobic.009G116700.v3.1, Sobic.001G079500.v3.1, Sobic.001G095700.v3.1, Sobic.007G077466.v3.1, Sobic.009G085100.v3.1, Sobic.004G286600.v3.1 and the other related genes. Related to Fig.5.

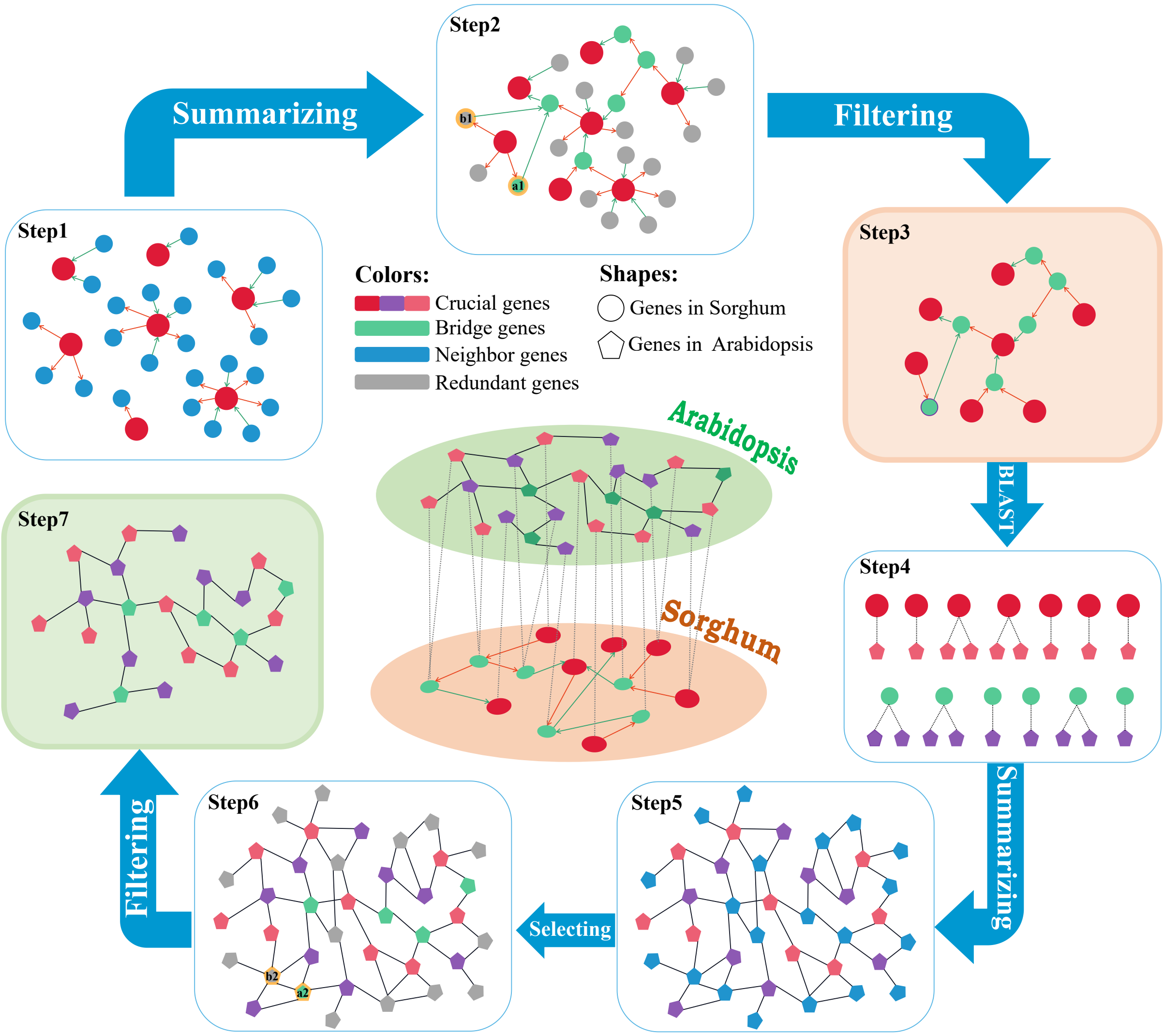

Figure S3: Schematic flowchart to construct Fig.5A in the main text. Detailed construction processes are as follows: Step1. Searching for the first-order neighbors of the crucial genes (red nodes) in *Sorghum* from PlantRegMap; Step2. Selecting bridge genes to connect the crucial genes. If there are several bridge genes, only the gene that most correlated with drought stress is retained. For example, suppose a1 and b1 are both bridge genes, and a1 involves in drought related processes, then we only consider the bridge gene a1; Step3. Integrating the regulatory relationships among the crucial genes and the selected bridge genes, and deleting other redundant genes, then the lower-layer of the bilayer network in Fig.5A is obtained; Step4. Finding homologous genes in *Arabidopsis*. For the genes that appear in step3, based on TAIR (https://www.arabidopsis.org), sequence alignment analysis is performed, and choosing genes with E value < 0.1 as homologous genes; Step5. Constructing primitive protein-protein interaction network among the homologous genes in *Arabidopsis*, which is based on the STRING database (https://cn.string-db.org). Adding possible bridge genes (blue nodes) to connect the homologous genes. Step6. Selecting bridge genes to simplify the network. Similar to Step2, among the added genes, those genes that are most correlated with drought stress are retained. For example, assume that gene a2 is more correlated with drought stress than gene b2, then a2 is retained and served as a bridge gene in the next step. Step7. Integrating the regulatory networks among the homologous genes and the selected bridge genes, the upper-layer of Fig.5A is obtained. Finally, by integrating the constructed networks in Step 3 and Step7, we obtain Fig.5A. Related to Fig.5.

Table S1: Results of sequence alignment analysis for the top-20 ranked genes in *Sorghum* with those in *Arabidopsis thaliana*. The E value is a statistical value related to the sequence alignment. Smaller E value indicates more reliable result. PMID denotes the PubMed unique identifier (https://pubmed.ncbi.nlm.nih.gov) for paper that studied the related genes. Related to Fig. 4 and Fig. 5.

Gene ID	Stage	Homologous gene	E value	GO terms(Homologous gene)	PMID
Sobic.002G424350.v3.1	Stage 1	AT4G24380	5.00×10^{-7}	”response to water deprivation”<br>”regulation of defense response”	34562334
Sobic.009G185800.v3.1	Stage 1	AT1G54290	2.00×10^{-73}	”response to water deprivation”	34562334
Sobic.006G173900.v3.1	Stage 1	AT2G43350	5.00×10^{-33}	”cellular response to water deprivation”	16998070
		AT2G31570	3.00×10^{-30}	”defense response”	34562334
Sobic.004G009600.v3.1	Stage 1	CHR12	9.00×10^{-12}	”response to water deprivation”	17605754
		INO80	1.50×10^{-2}	”regulation of transcription from RNA polymerase II promoter in response to stress”	17986450
Sobic.002G268100.v3.1	Stage 1	PMH1	3.00×10^{-6}	”response to water deprivation”	28649253, 18725370
		STRS2	5.00×10^{-3}	”response to water deprivation”	17556511, 25517295, 18725370
Sobic.001G517600.v3.1	Stage 1	AT5G23950	2.50×10^{-2}	”defense response”<br>”response to water deprivation”	34562334
Sobic.004G058600.v3.1	Stage 1	ALDH10A8	7.00×10^{-6}	”response to water deprivation”	21053011
Sobic.003G229400.v3.1	Stage 1	MPK3	6.00×10^{-27}	”priming of cellular response to stress”	19318610, 30977137
		MPK6	2.00×10^{-25}	–	22975351, 28857351, 24942184, 25635681
Sobic.001G405500.v3.1	Stage 1	PDC1	0	–	29777132
Sobic.009G005900.v3.1	Stage 1	ACT7	0	–	31035927
Sobic.009G171400.v3.1	Stages 1 & 2	AT1G59950	9.30×10^{-2}	”response to water deprivation”	34562334
Sobic.006G011300.v3.1	Stage 2	AT4G14500	2.00×10^{-42}	”response to water deprivation”	34562334
Sobic.009G116700.v3.1	Stage 2	XERO1	2.00×10^{-12}	”response to water deprivation”	17986450
		RAB18/ATDI8	7.00×10^{-12}	”response to water”<br>”response to water deprivation”	7823904
		LTI45/ERD10	6.00×10^{-7}	”response to water”<br>”response to water deprivation”	20054552
		LEA	2.00×10^{-6}	”response to water deprivation”	17986450
		RD17/COR47	7.00×10^{-6}	”response to water deprivation”	11779861
		LTI30/XERO2	8.00×10^{-5}	”response to water deprivation”	7948863
		ERD14	8.00×10^{-5}	”response to water deprivation”	17986450
Sobic.010G008600.v3.1	Stage 2	SOS6	2.00×10^{-169}	”response to water deprivation”	20409003
		LEW2	3.00×10^{-27}	”response to water deprivation”	15998313
		MRE1	4.00×10^{-26}	”defense response”	16255250
Sobic.002G374100.v3.1	Stage 2	AT2G25460	6.80×10^{-2}	”response to water deprivation”	34562334
Sobic.009G023200.v3.1	Stage 2	GPRP3	7.00×10^{-7}	”response to water deprivation”	34562334
Sobic.002G207300.v3.1	Stage 2	CPR5	7.90×10^{-2}	”defense response”	11437443, 24603484, 21556325, 22963672
Sobic.005G139700.v3.1	Stage 2	RBOHD	3.00×10^{-153}	–	35163000, 32695131
Sobic.001G407600.v3.1	Stage 2	FAD8	1.00×10^{-167}	–	34777447, 16236147
		FAD3	1.00×10^{-103}	–	24368335, 20953666
Sobic.004G052000.v3.1	Stage 2	PBS1	4.00×10^{-7}	–	29127881
		CRK4	2.00×10^{-5}	–	27406784
Sobic.003G081900.v3.1	Stages 2 & 3	HSP17.4	7.00×10^{-44}	”response to abiotic stimulus”	31261879
Sobic.001G078700.v3.1	Stage 3	DAG2	3.00×10^{-8}	”cellular response to water stimulus”	25850831
Sobic.006G098500.v3.1	Stage 3	GRP2	1.00×10^{-2}	”response to water deprivation”	16207746, 24268168
Sobic.007G077466.v3.1	Stage 3	WRKY75	4.00×10^{-4}	”regulation of DNA-templated transcription in response to stress”	17322336, 24146023, 31349781, 30231862
		WRKY66	1.00×10^{-3}	”response to water”	34562334, 29079899
Sobic.009G237600.v3.1	Stage 3	GBF3	2.00×10^{-11}	–	28831141, 30081325
Sobic.006G219300.v3.1	Stage 4	MRP4	1.10×10^{-2}	”response to water deprivation”	15225287, 30288712
Sobic.009G085100.v3.1	Stage 4	IAA7/AXR2	3.00×10^{-19}	”response to water deprivation”	32689451, 24992887, 8979397
Sobic.009G132900.v3.1	Stages 4 & 5	PPDK	0	”response to water deprivation”	34562334
Sobic.003G190100.v3.1	Stages 4 & 5	ALPHAVPE	2.00×10^{-126}	”defense response”	34562334
		BETAVPE	3.00×10^{-61}	”response to water deprivation”	34562334
Sobic.001G498000.v3.1	Stage 5	ARSK1	1.00×10^{-70}	”response to water”	7655506, 34562334
		AT1G74490	1.00×10^{-38}	”defense response”	34562334
		PBS1	2.00×10^{-31}	”defense response”	11359614
		AT1G07870	9.00×10^{-22}	”response to water deprivation”	34562334
		GPK1	2.00×10^{-11}	”response to water deprivation”	34562334
		AT3G59350	3.00×10^{-9}	”response to water deprivation”	34562334
		AT3G17410	1.00×10^{-7}	”positive regulation of response to water deprivation”	29970817
Sobic.004G286600.v3.1	Stage 5	ERD14	2.00×10^{-9}	”response to water deprivation”	18359842,17986450
		RD17/COR47	3.00×10^{-8}	”response to water deprivation”	11779861
		XERO1	9.00×10^{-8}	”response to water deprivation”	17986450
		RAB18/ATDI8	9.00×10^{-8}	”response to water deprivation”	7823904
		LTI45/ERD10	4.00×10^{-6}	”response to water deprivation”	17986450
		AT3G09540	2.00×10^{-3}	”response to water deprivation”	34562334
		LEA	2.00×10^{-3}	”response to water deprivation”	17986450
